# Case report: lady with bone pains for 5 years—parathyroid carcinoma

**DOI:** 10.1186/s13104-018-3711-0

**Published:** 2018-08-29

**Authors:** Azra Rizwan, Abid Jamal, Maseeh Uzzaman, Saira Fatima

**Affiliations:** 1 Section of Endocrinology, Department of Medicine, Aga Khan University Hospital, Stadium Road, P.O. Box 3500, Karachi, 74800 Pakistan; 2 Department of Surgery, Healthcare Hospital, Defense Housing Authority, Phase 1, Karachi, Pakistan; 3 Section of Nuclear Medicine, Department of Radiology, Aga Khan University Hospital, Stadium Road, P.O. Box 3500, Karachi, 74800 Pakistan; 4 Section of Histopathology, Department Pathology, Aga Khan University Hospital, Stadium Road, P.O Box 3500, Karachi, 74800 Pakistan

**Keywords:** Parathormone (PTH), Sestamibi scan, Hypercalcemia, Primary hyperparathyroidism, Case report

## Abstract

**Background:**

Parathyroid cancer is a rare cause of primary hyperparathyroidism. It presents a diagnostic and therapeutic challenge that may not be recognized preoperatively, and is often not conclusively identified during the operation. We present the case of a lady with backache and hypercalcemia, but with inadequate work-up for her condition for several years.

**Case presentation:**

A middle aged lady of Asian descent presented with backache. Initial work up revealed mild hypercalcemia, negative work up for multiple myeloma, negative sestamibi scan for parathyroid pathology. A phenomenally elevated parathormone (PTH) level—2105 pg/mL (16–87 pg/mL), and rising serum calcium, 15.1 mg/dL, (8.6–10.5 mg/dL), ordered years later prompted a repeat sestamibi scan and ultrasonography of neck. Based on these investigations, a diagnosis of primary hyperparathyroidism, with high suspicion of parathyroid cancer was made. The patient underwent surgical tumour resection, with subsequent histopathological confirmation of diagnosis.

**Conclusion:**

In the setting of hypercalcemia, PTH level assessment is a must. This helps to differentiate between the parathyroid dependant and independent causes of high serum calcium, thereby encouraging a comprehensive pathway to the work up of the cause of hypercalcemia. The parathyroid cancer is a very rare cause of hypercalcemia, which needs to be considered in the differentials of primary hyperparathyroidism, particularly in the setting of high PTH levels.

**Electronic supplementary material:**

The online version of this article (10.1186/s13104-018-3711-0) contains supplementary material, which is available to authorized users.

## Background

Primary hyperparathyroidism affects approximately 1% of the world’s adult population [1]. Single parathyroid adenomas constitute the majority of cases (80–85%). Most of the remaining cases are due to hyperplasia of multiple parathyroid glands [1–3], with parathyroid cancer being a rare cause (1–2%) [1–3]. Parathyroid carcinoma presents a diagnostic and therapeutic challenge as it is a rare endocrine malignancy, which may not be recognized preoperatively, and often is not conclusively identified during the operation [1].

Patients with parathyroid carcinoma usually present with a severe form of hyperparathyroidism at the time of diagnosis including bone disease, renal disease, neurologic manifestations and gastrointestinal symptomatology, compared to the relatively asymptomatic presentation of benign parathyroid disease [1, 4]. However, diagnosis is sometimes made after routine biochemical screening has revealed hypercalcemia in an asymptomatic individual who is then found to have raised PTH [1, 4, 5].

The etiology of parathyroid carcinoma is unclear, although it has been associated with neck radiation in sporadic cases [1]. Carcinomas have also been reported in familial hyperparathyroidism, such as hereditary hyperparathyroidism jaw tumor (HPT-JT) syndrome, which carries an increased risk of parathyroid cancer (15% vs 0.1% of sporadic parathyroid cancer) [6].

This case report describes a patient presenting with nonspecific bone pains, whose initial workup showed mild hypercalcemia, negative work up for multiple myeloma and a negative sestamibi scan for parathyroid pathology. A phenomenally elevated PTH level and rising serum calcium ordered years later prompted us to request for a repeat sestamibi scan and ultrasonography of neck. Based on these levels & radiological tests, a diagnosis of primary hyperparathyroidism, with high suspicion of parathyroid cancer, was made. Following this, the patient underwent surgical tumor resection, with subsequent histopathological confirmation of the diagnosis.

## Case presentation

A 53 year old Pakistani lady presented to the Medicine clinic of a local hospital in 2004 with a history of heel pain and lower back pain for 5 months. In this period, the patient had sustained a rib fracture and left humeral fracture. There was no history of diabetes, hypertension or any other chronic disease. She had not been on any form of medication, including steroids and traditional drugs widely available and prescribed in the region, prior to the onset of pain. At the time of the fractures, she had been placed on non steroidal anti inflammatory agents, acetaminophen and tramadol. There was no history of illicit drug use and she was a non smoker. Family history was unremarkable, particularly in the context of bone disease, and malignancy.

**Fig. 1:**
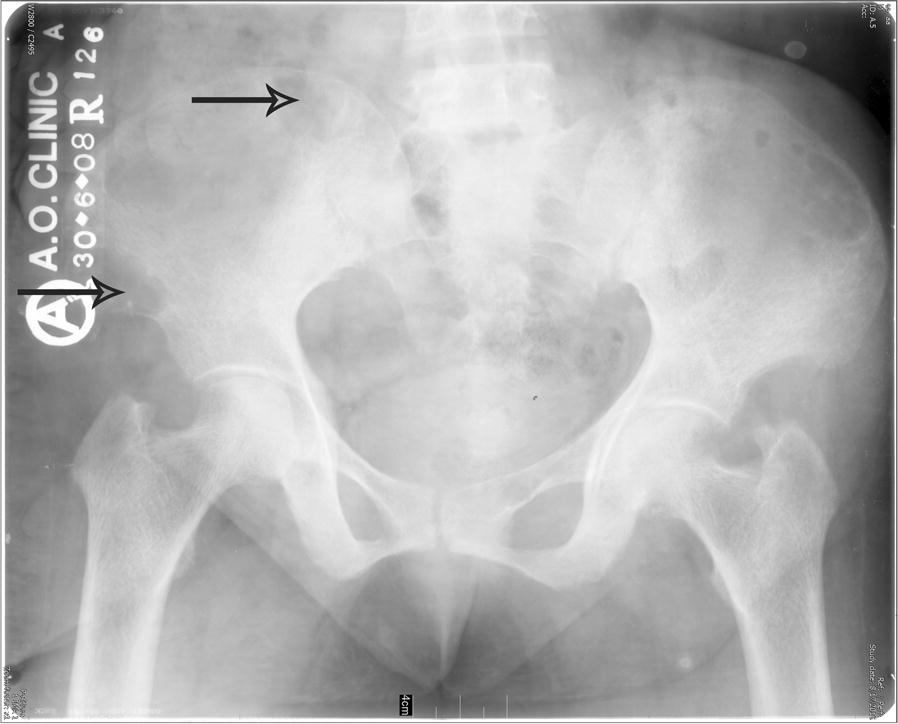
X-ray pelvis (punched out lytic lesions in right iliac bone)

Initial laboratory investigations had shown a mildly elevated total calcium level of 10.8 mg/dL {2.7 mmol/L}-(no albumin level result available from that time for correction). Parathormone levels (PTH) had not been determined. There was no vitamin d or renal function report available from that time. X-Ray pelvis revealed lytic lesions in the right iliac bone (Fig. 1). A magnetic resonance imaging (MRI) of the lumbosacral spine showed some signal changes. The differentials based on the MRI were metastatic bone disease or multiple myeloma.

Serum protein electrophoresis was normal. The patient then got lost to follow-up. Her work up was resumed 4 years later when her bone pains had started flaring up. Bone marrow examinations done back in 2007, and later in 2009, were negative for multiple myeloma. A bone scan in November 2009 showed generalized increased tracer uptake over the skull and both the axial and appendicular skeletons- findings in favor of metabolic bone disease (Fig. 4a). An initial planar parathyroid sestamibi scan requested by a general practitioner in November 2009 was negative for any functioning parathyroid adenoma in the neck or superior mediastinum. No serum PTH report was available from this time either. Following this workup, the patient was treated empirically for bone pains with calcium supplements, an empiric vitamin d injection, and intravenous zoledronic acid 5 mg (without prior bone mineral density assessment via DXA scan). This empiric treatment was instituted by an orthopedic surgeon whom she had been referred to. The patient experienced only a slight improvement in bone pains with this treatment and also developed nausea, vomiting and anorexia. Subsequently, she sought care at the National Institute of Diabetes and Endocrinology, Dow University Health Sciences, Karachi, Pakistan.

At presentation, the patient was well oriented and of functional class 3 (wheel chair bound, able to walk only with support). Her blood pressure was 110/70 mmHg. Neck examination revealed no mass or lymphadenopathy. She had a significant proximal myopathy as well as curved thighs. She had shortened fingers, and spinal scoliosis was evident. Severe generalized bone tenderness was elicited. There was no focal deficit. Laboratory investigations at this time showed a calcium level of 15.1 mg/dL{3.775 mmol/L}, (corrected for albumin of 3.6 mg/dL{36 g/L}); Vitamin D3 level of 33.92 ng/mL{84.664 nmol/L}; phosphorus 2.3 mg/dL {0.743 mmol/L}and alkaline phosphatase of 1298 IU/L {21.633 µkat/L}. Her 24 h urine calcium was 155 mg/day {3.875 mmol/day}, with urine calcium to creatinine ratio of 0.02. Her creatinine level was 1.3 mg/dL {114.92 µmol/L}(Table 1). The estimated glomerular filtration rate (calculated through Cockcroft-Gault equation) was 50 mL/min {0.835 mL/second).

**Table 1:** Laboratory work up at presentation to Endocrine Institute—5 years after onset of backache

Laboratory investigations	Normal ranges
Repeat corrected calcium 15.1 mg/dL	{8.6–10.5}
Phosphorus 2.3 mg/dL	{2.7–4.8}
Alkaline phosphatase 1298 IU/L	{29–132}
Parathormone 2105 pg/mL	{16–87}
25 OH vitamin D 33.92 ng/mL (following intramuscular administration of vitamin D)	{> 30}
Serum Creatinine 1.3 mg/dL	{0.6–1.35}
24 h urine calcium 155 mg/day	{100–300}

**Fig. 2:**
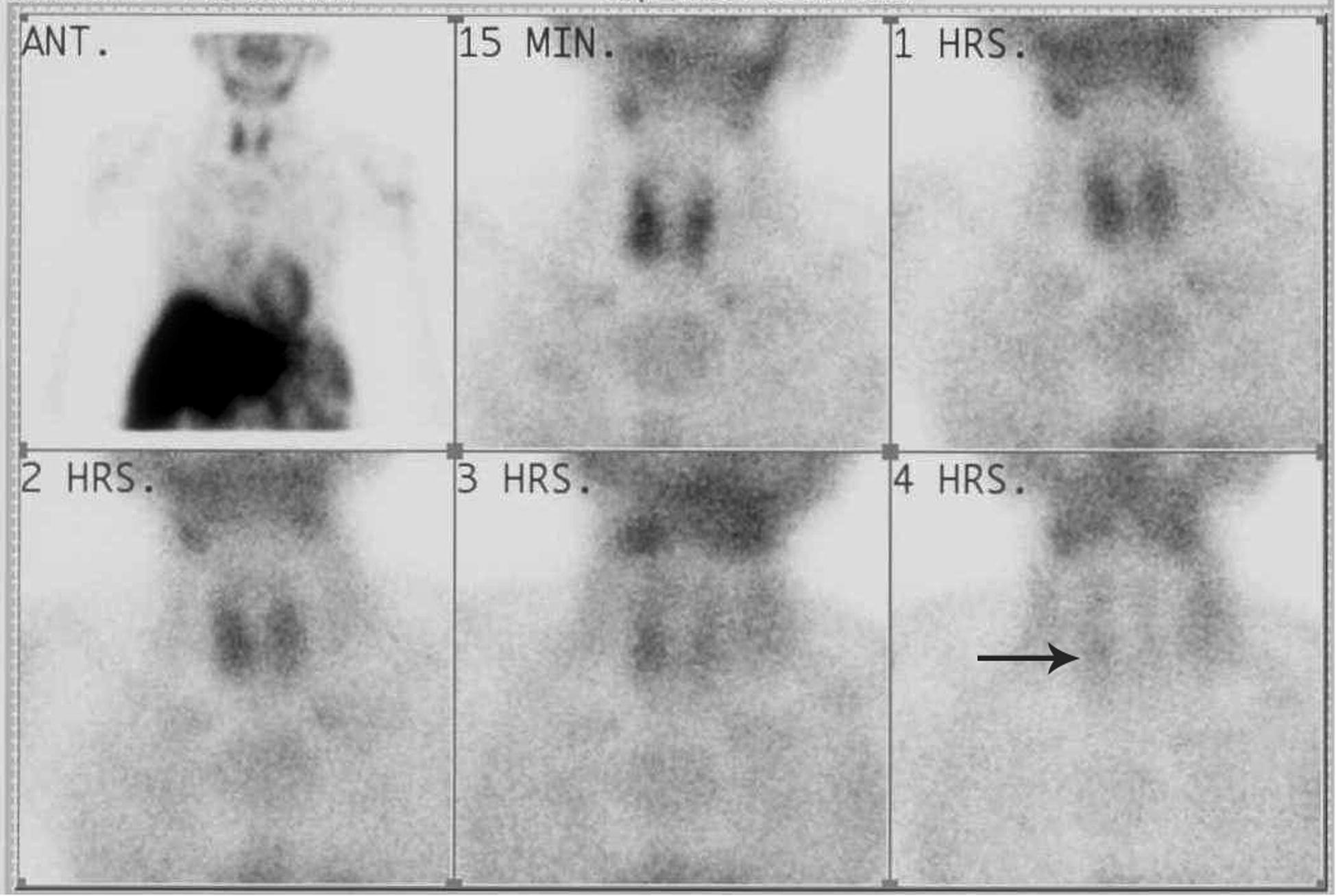
Parathyroid sestamibi scan (areas of tracer retention over upper and lower poles of right lobe thyroid-findings highly suggestive of primary hyperparathyroidism)

Following these tests, the patient’s PTH level was ordered and determined to be 2105 pg/mL {2105 ng/L} [Table 1]. Ultrasonography of the neck showed a solid hypo echoic, well-circumscribed mass lesion, measuring 1.8 × 1.2 cm at the lower pole of the right lobe of thyroid. There were no calcifications or lymphadenopathy. Appearances were suggestive of parathyroid adenoma. Both lobes of the thyroid appeared normal. A repeat planar sestamibi scan, (requested from a different institute in the city), revealed areas of tracer retention over upper and lower poles of the right lobe of thyroid. The intensity of retained tracer was more over the right inferior parathyroid gland. The findings were highly suggestive of hyperparathyroidism (Fig. 2).

A bone mineral density scan showed a T score of − 2.9 in the spine, − 3.8 in the hip and − 4.5 in the distal forearm, consistent with severe osteoporosis. The Z scores at the spine, hip and distal forearm were − 2.0, − 3.1 and − 3.6, respectively (Table 2).

**Table 2:** Bone mineral density (BMD), T score and Z scores, prior to and after parathyroidectomy

Scan date	02/12/2009 (pre operative)	11/01/2011 (post operative)
BMD hip (g/cm^2^)◦	0.498	0.886
T score hip	− 3.6	− 0.5
Z score hip	− 3.1	0.2
BMD spine (g/cm^2^)◦	0.729	0.933
T score spine	− 2.9	− 1.0
Z score spine	− 2.0	− 0.0
BMD forearm (g/cm^2^)◦	0.333	0.402
T score forearm	− 4.5	− 3.2
Z score forearm	− 3.6	− 2.2

◦BMD values at our institute (AKUH) for DXA done on 2.12.2009 were compared with the study of 11.01.2011. The comparative analysis showed statistically significant improvement in BMD values. The change in BMD was greater than the Least Significant Change (LSC) of our Department at AKUH for each region of interest. Therefore, it was considered significant (LSC for AKUH is: Hip: 0.030; Spine: − 0.034; Forearm: 0.043)

As per Institute protocol and World Health Organization (WHO) guidelines BMD of only the non-dominant hip (in this patient’s case, the right hip) was measured, (position statement of International Society for Clinical Densitometry, attached as Additional file 1)

Ultrasonography of the kidneys revealed a single renal stone (0.6 cm) and no neprocalcinosis.

**Fig. 3:**
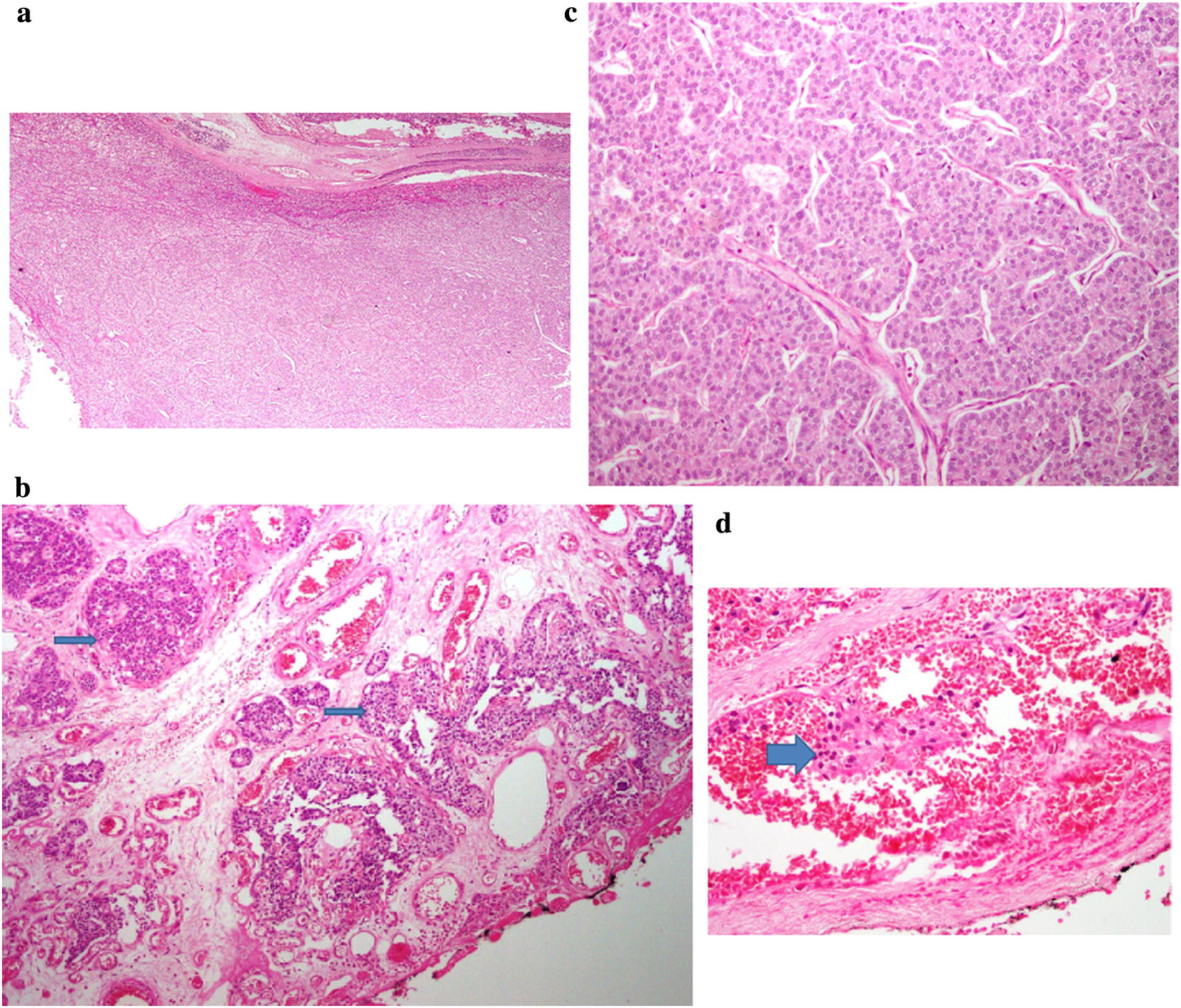
**a** (H&E, ×40): circumscribed parathyroid tumour composed of nests of polygonal cells with richly vascular stroma. **b** (H&E, ×100): tumour cells infiltrating adjacent soft tissue. **c** (H&E, ×200): high power view of tumour showing polygonal cells exhibiting nuclear atypia and scattered mitoses. **d** (H&E, ×400): tumour nests invading the blood vessel

Based on the biochemistry results of hypercalcemia, associated with elevated PTH levels, a diagnosis of primary hyperparathyroidism was made. Subsequent sestamibi scan and neck imaging facilitated us to localize the abnormal parathyroid gland. The DXA scan was useful for evaluation of the bone mineral density. In view of the phenomenally high levels of parathyroid hormone, (more than 10 times upper limit of normal), the pre-operative suspicion of parathyroid cancer was high [1, 7]. The patient was rehydrated with intravenous fluids. Subcutaneous calcitonin injections at a dose of 4 units/kg every 12 h were administered to tide her over until the surgery. Once her calcium levels had come down to 10.5 mg/dL {2.625 nmol/L}L, she was operated upon. At surgery, right hemithyroidectomy and inferior parathyroidectomy with level six lymph node resection was done. The lymphadenectomy was performed as there was evidence of enlarged lymph nodes at neck exploration. The size of the lesion was measured as 2.5 × 1.5 × 1 cm. Histopathology showed features consistent with parathyroid cancer (Fig. 3a–d). Capsular invasion and focal vascular invasion were noted. However, margins of excision were tumor free. The excised lymph nodes did not show evidence of tumour infiltration. The patient was not given external radiation therapy postoperatively. Literature review revealed that post operative adjuvant radiation therapy may only have a role in the management of patients with a histologically positive margin following en bloc resection, or in those with lymph node metastases [1, 4, 8].

Postoperative PTH level, performed on the second day of surgery, was 59 pg/mL {59 ng/L} (16–87). On the third postoperative day, the patient’s serum corrected calcium declined to 6 mg/dL {1.5 mmol/L}. This was associated with paresthesias around her mouth and carpo-pedal spasm. There were no seizures, although there was some confusion in terms of time and place. Intravenous calcium (2 g calcium gluconate, equivalent to 180 mg elemental calcium, in 50 mL 5% dextrose water) was infused over 20 min. Re-monitoring of calcium levels revealed persistent hypocalcemia. A slow infusion of calcium was initiated at an initial rate of 50 mL/h. This was prepared by adding 100 mL of 10% calcium gluconate (equivalent to 900 mg elemental calcium) to 1000 mL 5% dextrose water. The infusion rate was adjusted, with a goal to maintain calcium levels at lower end of normal range. On the fifth post-operative day, the calcium level had risen to 9.0 mg/dL {2.25 nmol/L}. Neurologic examination was normal and she was tolerating oral diet. Oral calcium supplementation was initiated (Qalsan D four times daily-equivalent to 2 g elemental calcium per day). She was discharged on oral calcium and vitamin D supplementation with active vitamin D, (calcitriol) 0.25 µg twice daily, in a stable condition.

**Fig. 4:**
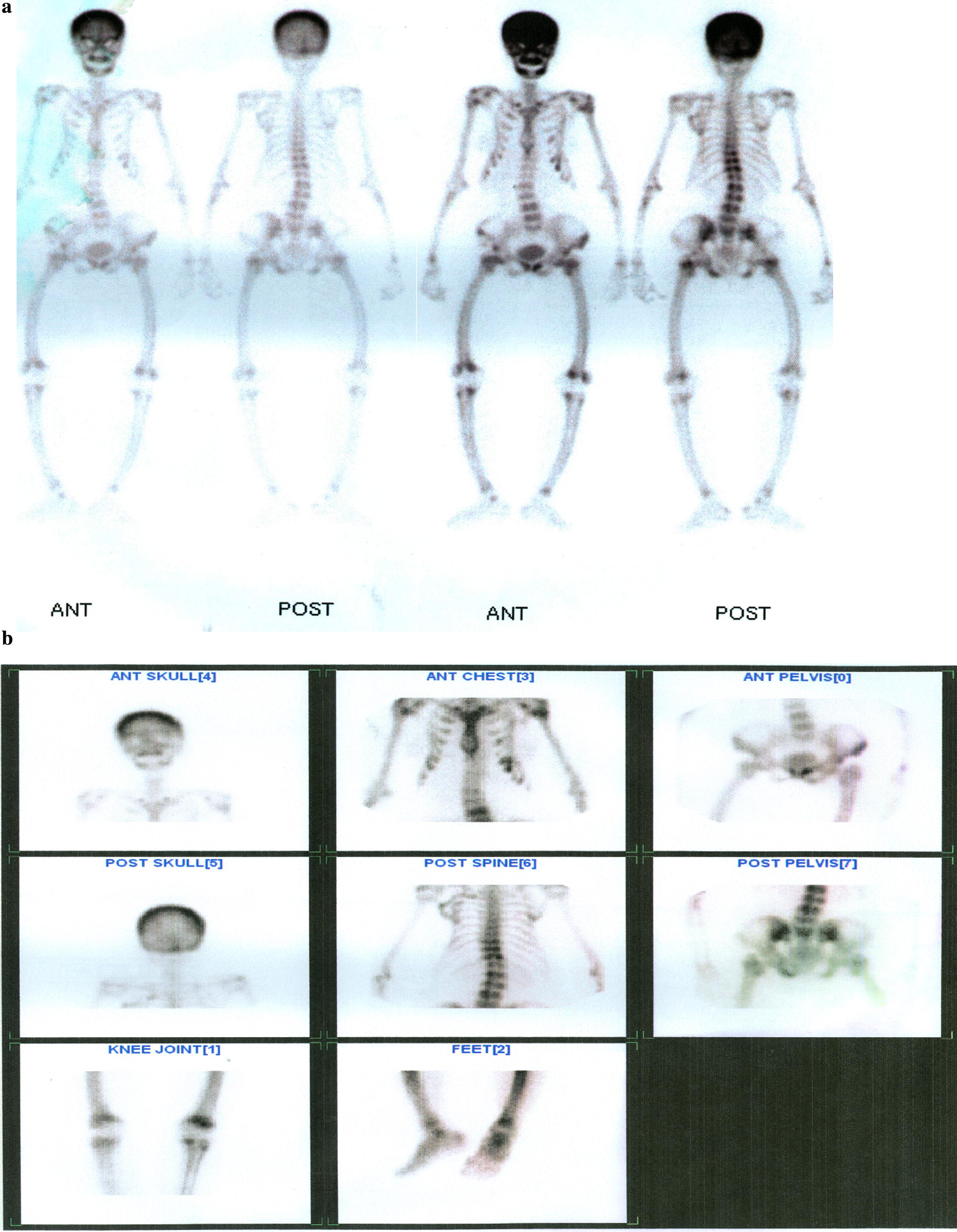
**a** Bone Scan prior to parathyroidectomy (10/11/2009): generalized increased tracer uptake over the skull, both axial and appendicular skeleton: findings in favour of metabolic bone disease. **b** Bone Scan 3 months after parathyroidectomy (18/02/2010): diffusely increased tracer uptake in axial and appendicular skeleton with increased bone to soft tissue tracer uptake ratio-findings consistent with metabolic bone disease. No appreciable change in scan pattern seen (from previous scan of 10/11/2009)

At follow-up, her appetite and mobility had improved significantly, although she continued to experience bone pains. Corrected calcium was 9.5 mg/dL {2.375 nmol/L}. A repeat skeletal scintigraphy done 3 months after parathyroidectomy did not demonstrate a significant change in the lytic lesions (Fig. 4a, b). A repeat DXA scan 2 years down the line revealed a significant improvement in bone mineral density at all sites, though more so at the spine and hip, than at the forearm (Table 2). Thereafter, we followed her clinically, as she was not keen to have further radiologic testing done. We have been monitoring her calcium and PTH levels on an annual basis. They have remained within their normal range till date (2018). She is now functional class 2, (no longer wheel chair bound), and on regular calcium and vitamin D supplements (patient perspective, attached as Additional file 2).

## Discussion and conclusion

This case highlights several unusual features of a very rare disease of the parathyroid gland—parathyroid cancer—documented to be a causative factor in less than 1% of cases of hyperparathyroidism [1–3]. Compared with patients with parathyroid adenomas, patients with parathyroid carcinomas are more likely to have symptoms, a neck mass, bone and kidney disease, marked hypercalcemia, very high serum parathyroid hormone (PTH) concentrations and, subsequently, high urinary calcium losses [1, 7]. In this patient, although the preoperative parathyroid hormone level was inexorably high at 2105 pg/mL (16–87), the 24 h urine calcium was not *as* high (155 mg/day) as expected in association with these levels of PTH [9–11], given a reasonably sufficient (840 mL) 24 h urine collection. The relatively low 24 h urine calcium could in part be explained by the reduction of renal function (creatinine clearance of 50 mL/min). The serum calcium levels were not as high as reported in the literature for parathyroid cancers [1, 7, 9]. The initial level was 10.8 mg/dL {2.7 mmol/L}. The highest level documented was 15.1 mg/dL{3.775 mmol/L}, that too only after she had been placed on calcium and vitamin d supplementation by an orthopedic surgeon, whom she had presented to for her bone pains. The orthopedic surgeon had probably been treating her as a regular case of primary osteoporosis, without a complete evaluation and consideration for the initial mildly elevated calcium of 10.8 mg/dL {2.7 mmol/L}. The possibility exists that her baseline vitamin D level, unfortunately not assessed prior to institution of parental vitamin d, may have been low. This may have led to the relatively lower baseline levels of calcium than expected in the context of parathyroid carcinoma. Unmasking of primary hyperparathyroidism subsequent to vitamin D replacement has been reported in the literature [10].

Ultrasonography of our patient revealed a 1.8 × 1.2 cm hypo echoic, though well-circumscribed solid lesion, with no calcifications or lymphadenopathy, at lower pole of the right lobe of the thyroid. Cetani et al. in their review state that mass size > 3 cm, marked hypoechogenicity, a lobulated non homogenous pattern, calcifications and degenerative changes may raise the suspicion of parathyroid cancer [1, 12]. Another unusual feature of this patient’s presentation was that the first sestamibi scan was negative. The repeat scan (Fig. 2) (requested from a different radiologic centre) showed *some* tracer retention over the upper and lower poles of right lobe of thyroid, which co related with findings obtained on ultrasonography. The latter had revealed a mass lesion at the lower pole of the right lobe of the thyroid, though no malignant characteristics, such as calcifications, degenerative changes or irregular halo sign [1, 12] had been reported. Although both scans were planar, the negative scan had been done using a low energy general purpose collimator (LEGP), while the repeat scan was done using a low-energy high resolution collimator (LEHR). The discrepancy between the results of the two sestamibi scans could have been due to these different techniques employed. Sestamibi scan does not distinguish a parathyroid adenoma from a parathyroid carcinoma [11]. Considering the phenomenally raised parathormone levels, however, a hot nodule had been anticipated. The majority of cases of parathyroid cancer reported in the literature have described a dramatic lighting up of the lesion on the sestamibi scan, with a sensitivity of 85–100%, and specificity of 100% [13]. In our case, there was subtle tracer retention over upper and lower poles of right lobe of thyroid, with intensity of retention higher over the inferior pole. A pin- hole collimator technique to obtain a magnified, higher resolution image focused on the neck, was not available at either Institute at that time. There has been a case reported in the literature of false negative scintigraphy in parathyroid carcinoma with associated brown tumours (term discussed below) [13].

From the neurologic and renal stand point the patient had been well preserved. There had been no manifestations of altered sensorium, even at the stage when her serum calcium had reached a level of 15.1 mg/dL {3.775 mmol/L}. Ultrasonography of her kidneys had revealed normal sized kidneys with intact corticomedullary differentiation, no nephrocalcinosis and a single small renal stone. In most cases of parathyroid carcinoma, central nervous system and renal manifestations are apparent at the time of diagnosis [1, 4]. In the case of this patient, the disease, though remaining undiagnosed for 5 years, seems to have involved predominantly the skeletal system, leading to severe osteoporosis. Her shortened fingers on physical examination were indicative of pharyngeal tuft resorption. The low Z scores of ≤− 2.0 at the spine, hip and forearm were highly suggestive of a secondary cause of osteoporosis. The bone mineral density at the forearm (T score − 4.5) was lower than at the spine (T score − 2.9) Table 2. This is in keeping with the fact that the parathormone has a greater impact on cortical bone (distal forearm and hip) than the spine, which consists of cancellous bone [12]. Correspondingly, a follow up dexa scan 2 years after the parathyroidectomy revealed a significant improvement in bone mineral density at all the sites, though a slower improvement was observed at the forearm (Table 2). This was likely related to the prolonged preoperative exposure to high levels of parathormone at the cortical bones of the forearm. The lytic lesions of the bones in primary hyperparathyroidism result from excess osteoclast activity leading to a collection of osteoclasts intertwined with fibrous tissue and undermineralized woven bone. Haemosiderin accumulation gives rise to the brown disclouration—hence originated the label of “brown tumours” for these lytic lesions [2, 12]. Correspondingly, as evident in this lady’s case, these lesions were originally interpreted as metastases or myeloma lesions radiologically. Both these conditions, though important differentials to consider, result in PTH-independent hypercalcemia, versus the PTH- dependant hypercalcemia ensuing from primary hyperparathyroidism.

Long-standing hypercalcemia resulting from autonomous parathormone production leads to suppression of the remaining parathyroid glands. Following parathyroidectomy, recovery of function has been variable, ranging from hours to a couple of weeks. In many cases, oral calcium supplementation alone adequately restores calcium levels. In some situations, as was the case in our patient, more severe hypocalcemia ensues, requiring intravenous calcium supplementation. As a result of PTH suppression, the 1-alfa hydroxylation of vitamin D, at the renal tubular level, is concurrently reduced. This necessitates supplementation with the active form of vitamin d. This condition has been described as the “hungry bone syndrome”, usually found to occur 3–5 days following parathyroidectomy. The reason for its development is thought to be due to significant increases in osteoblastic function, bone formation and deposition of calcium and phosphorus in previously deprived bones [2, 14, 15]. Our patient had a long-standing history of primary hyperparathyroidism, phenomenally elevated PTH levels, marked hypercalcemia and elevated alkaline phosphatase levels (indicating high bone resorption). She also had radiologic signs of osteitis fibrosa cystica (Fig. 1). These hallmarks very likely led to development of the typical hungry bone syndrome in this case. Concomitant with her long-standing history of hyperparathyroidism, the lytic lesions on her follow-up bone scintigraphy, done 3 months following parathyroidectomy, did not show any significant improvement.

A number of important lessons can be learned from this case. In the setting of bone pains and hypercalcemia, the parathormone level, along with a vitamin D assessment and renal function tests, should have been checked when the patient had first presented to the general practitioner back in 2004. This would have differentiated between parathyroid-dependant and parathyroid-independent hypercalcemia, and may have avoided an exhaustive work up for multiple myeloma. The patient had previously undergone bone marrow examinations on two occasions, both showing a normal result. The physicians may have been misguided by the only two differentials suggested on the MRI Lumbosacral spine report: “Multiple Myeloma versus metastasis.” In both these conditions, parathyroid levels are suppressed in the setting of hypercalcemia. Unfortunately, the parathormone level and relevant testing, including vitamin D and renal function tests, were only requested 5 years down the line when the patient first presented to the Endocrinologist. This led to an unacceptable delay in the diagnosis. It is unclear why the first sestamibi scan was requested, without a prior parathyroid hormone level assessment, at the time when the patient was following up elsewhere for her condition.

In the setting of hypercalcemia, parathyroid hormone level assessment is a must, to differentiate between the parathyroid dependant and independent causes of high serum calcium, thereby encouraging a comprehensive pathway to the work-up of the cause of the hypercalcemia. The Parathyroid carcinoma is a very rare cause of hypercalcemia that can present in several ways and which needs to be considered in the differentials of primary hyperparathyroidism, particularly in the setting of high parathyroid hormone levels.

## Additional files

**Additional file 1.** ISCD OF STAT 2015. Title of Data: International Society for Clinical Densitometry Position Statement, updated 2015. Description of Data: describes indications for BMD testing, Reference database for Tscores, and Serial BMD testing using concept of least significant change (LSC).

**Additional file 2.** Para cancer patient perspective. Title of Data: Patient Perspective. Description of Data: describes the patient’s view on how challenging the diagnosis of parathyroid cancer had been for her and her family, and the toll it took on them. She emphasizes the importance of creating awareness about the condition amongst general practitioners to expedite early referral to concerned speciality.

## Abbreviations

PTH: parathormone
DXA: dual energy X-ray absorptiometry
LEGP: low energy general purpose
LEHR: low energy high resolution
MRI: magnetic resonance imaging
