# Supplementary material for: Case report: lady with bone pains for 5 years—parathyroid carcinoma
Source: BMC Res Notes. 2018 Aug 29;11:617. doi: 10.1186/s13104-018-3711-0 (PMC6114890; doi:10.1186/s13104-018-3711-0)
Supplement: Supplementary file 1 — Additional file 1. ISCD OF STAT 2015. Title of Data: International Society for Clinical Densitometry Position Statement, updated 2015. Description of Data: describes indications for BMD testing, Reference database for Tscores, and Serial BMD testing using concept of least significant change (LSC). [file 13104_2018_3711_MOESM1_ESM.pdf]

ISCD

The International Society
For Clinical Densitometry

These are the Adult Official Positions of the ISCD as updated in 2015.The Official Positions that are new or revised since 2013 are in **bold type**.

Indications for Bone Mineral Density (BMD) Testing

- Women aged 65 and older
- For post-menopausal women younger than age 65 a bone density test is indicated if they have a risk factor for low bone mass such as;
  - Low body weight
  - Prior fracture
  - High risk medication use
  - Disease or condition associated with bone loss.
- Women during the menopausal transition with clinical risk factors for fracture, such as low body weight, prior fracture, or high-risk medication use.
- Men aged 70 and older.
- For men < 70 years of age a bone density test is indicated if they have a risk factor for low bone mass such as;
  - Low body weight
  - Prior fracture
  - High risk medication use
  - Disease or condition associated with bone loss.
- Adults with a fragility fracture.
- Adults with a disease or condition associated with low bone mass or bone loss.
- Adults taking medications associated with low bone mass or bone loss.
- Anyone being considered for pharmacologic therapy.
- Anyone being treated, to monitor treatment effect.
- Anyone not receiving therapy in whom evidence of bone loss would lead to treatment.

Women discontinuing estrogen should be considered for bone density testing according to the indications listed above.

Reference Database for T-Scores

- Use a uniform Caucasian (non-race adjusted) female normative database for women of all ethnic groups.*
- Use a uniform Caucasian (non-race adjusted) female reference for men of all ethnic groups *
- Manufacturers should continue to use NHANES III data as the reference standard for femoral neck and total hip T-scores.
- Manufacturers should continue to use their own databases for the lumbar spine as the reference standard for T-scores
- If local reference data are available they should be used to calculate only Z-scores but not T-scores.

*Note: Application of recommendation may vary according to local requirements.

Central DXA for Diagnosis

- The WHO international reference standard for osteoporosis diagnosis is a T-score of -2.5 or less at the femoral neck.
    - The reference standard from which the T-score is calculated is the female, white, age 20-29 years, NHANES III database
- Osteoporosis may be diagnosed in postmenopausal women and in men age 50 and older if the T-score of the lumbar spine, total hip, or femoral neck is -2.5 or less:*
    - In certain circumstances the 33% radius (also called 1/3 radius) may be utilized

*Note: Other hip regions of interest, including Ward's area and the greater trochanter, should not be used for diagnosis. Application of recommendation may vary according to local requirements.

- Skeletal sites to measure
    - Measure BMD at both the PA spine and hip in all patients
    - Forearm BMD should be measured under the following circumstances:
        - Hip and/or spine cannot be measured or interpreted.
        - Hyperparathyroidism
        - Very obese patients (over the weight limit for DXA table)
- Spine Region of Interest (ROI)
    - Use PA L1-L4 for spine BMD measurement
    - Use all evaluable vertebrae and only exclude vertebrae that are affected by local structural change or artifact. Use three vertebrae if four cannot be used and two if three cannot be used

    - BMD based diagnostic classification should not be made using a single vertebra.
    - If only one evaluable vertebra remains after excluding other vertebrae, diagnosis should be based on a different valid skeletal site
    - Anatomically abnormal vertebrae may be excluded from analysis if:
        - They are clearly abnormal and non-assessable within the resolution of the system; or
        - There is more than a 1.0 T-score difference between the vertebra in question and adjacent vertebrae
    - When vertebrae are excluded, the BMD of the remaining vertebrae is used to derive the T-score
    - The lateral spine should not be used for diagnosis, but may have a role in monitoring
- Hip ROI
    - Use femoral neck, or total proximal femur whichever is lowest.
    - BMD may be measured at either hip
    - There are insufficient data to determine whether mean T-scores for bilateral hip BMD can be used for diagnosis
    - The mean hip BMD can be used for monitoring, with total hip being preferred
- Forearm ROI
    - Use 33% radius (sometimes called one-third radius) of the non-dominant forearm for diagnosis. Other forearm ROI are not recommended

Fracture Risk Assessment

- A distinction is made between diagnostic classification and the use of BMD for fracture risk assessment.
- For fracture risk assessment, any well-validated technique can be used, including measurements of more than one site where this has been shown to improve the assessment of risk.

Use of the Term “Osteopenia”

- The term “osteopenia” is retained, but “low bone mass” or “low bone density” is preferred.
- People with low bone mass or density are not necessarily at high fracture risk.

BMD Reporting in Postmenopausal Women and in Men Age 50 and Older

- T-scores are preferred.
- The WHO densitometric classification is applicable.

BMD Reporting in Females Prior to Menopause and in Males Younger Than Age 50

- Z-scores, not T-scores, are preferred. This is particularly important in children.
- A Z-score of -2.0 or lower is defined as “below the expected range for age”, and a Z-score above -2.0 is “within the expected range for age.”
- Osteoporosis cannot be diagnosed in men under age 50 on the basis of BMD alone.
- The WHO diagnostic criteria may be applied to women in the menopausal transition.

Z-Score Reference Database

- Z-scores should be population specific where adequate reference data exist. For the purpose of Z-score calculation, the patient’s self-reported ethnicity should be used.

Serial BMD Measurements

- Serial BMD testing can be used to determine whether treatment should be started on untreated patients, because significant loss may be an indication for treatment.
- Serial BMD testing can monitor response to therapy by finding an increase or stability of bone density.
- Serial BMD testing can evaluate individuals for non-response by finding loss of bone density, suggesting the need for reevaluation of treatment and evaluation for secondary causes of osteoporosis.
- Follow-up BMD testing should be done when the expected change in BMD equals or exceeds the least significant change (LSC).
- Intervals between BMD testing should be determined according to each patient’s clinical status: typically one year after initiation or change of therapy is appropriate, with longer intervals once therapeutic effect is established.
- In conditions associated with rapid bone loss, such as glucocorticoid therapy, testing more frequently is appropriate.

Phantom Scanning and Calibration

The Quality Control (QC) program at a DXA facility should include adherence to manufacturer guidelines for system maintenance. In addition, if not recommended in the manufacturer protocol, the following QC procedures are advised:

- Perform periodic (at least once per week) phantom scans for any DXA system as an independent assessment of system calibration.

- Plot and review data from calibration and phantom scans.
- Verify the phantom mean BMD after any service performed on the densitometer.
- Establish and enforce corrective action thresholds that trigger a call for service.
- Maintain service logs.
- Comply with government inspections, radiation surveys and regulatory requirements.

Precision Assessment

- Each DXA facility should determine its precision error and calculate the LSC.
- The precision error supplied by the manufacturer should not be used.
- If a DXA facility has more than one technologist, an average precision error combining data from all technologists should be used to establish precision error and LSC for the facility, provided the precision error for each technologist is within a pre-established range of acceptable performance.
- Every technologist should perform an in vivo precision assessment using patients representative of the clinic's patient population.
- Each technologist should do one complete precision assessment after basic scanning skills have been learned (e.g., manufacturer training) and after having performed approximately 100 patient-scans.
- A repeat precision assessment should be done if a new DXA system is installed.
- A repeat precision assessment should be done if a technologist's skill level has changed.
- To perform a precision analysis:
    - Measure 15 patients 3 times, or 30 patients 2 times, repositioning the patient after each scan
    - Calculate the root mean square standard deviation (RMS-SD) for the group
    - Calculate LSC for the group at 95% confidence interval
- The minimum acceptable precision for an individual technologist is:
    - Lumbar Spine: 1.9% (LSC=5.3%)
    - Total Hip: 1.8% (LSC=5.0%)
    - Femoral Neck: 2.5% (LSC=6.9%)
    - Retraining is required if a technologist's precision is worse than these values
- Precision assessment should be standard clinical practice. Precision assessment is not research and may potentially benefit patients. It should not require approval of an institutional review board. Adherence to local radiologic safety regulations is necessary. Performance of a precision assessment requires the consent of participating patients.

Cross-Calibration of DXA Systems

- When changing hardware, but not the entire system, or when replacing a system with the same technology (manufacturer and model), cross-calibration should be performed by having one technologist do 10 phantom scans, with repositioning, before and after hardware change.
    - If a greater than 1% difference in mean BMD is observed, contact the manufacturer for service/correction
- When changing an entire system to one made by the same manufacturer using a different technology, or when changing to a system made by a different manufacturer, one approach to cross-calibration is:
    - Scan 30 patients representative of the facility's patient population once on the initial system and then twice on the new system within 60 days
    - Measure those anatomic sites commonly measured in clinical practice, typically spine and proximal femur
    - Facilities must comply with locally applicable regulations regarding DXA
    - Calculate the average BMD relationship and LSC between the initial and new machine using the ISCD DXA Machine Cross-Calibration Tool (www.ISCD.org)
    - Use this LSC for comparison between the previous and new system. Inter-system quantitative comparisons can only be made if cross-calibration is performed on each skeletal site commonly measured
    - Once a new precision assessment has been performed on the new system, all future scans should be compared to scans performed on the new system using the newly established intra-system LSC
- If a cross-calibration assessment is not performed, no quantitative comparison to the prior machine can be made. Consequently, a new baseline BMD and intra-system LSC should be established.

BMD Comparison Between Facilities

- It is not possible to quantitatively compare BMD or to calculate a LSC between facilities without cross-calibration.

Vertebral Fracture Assessment Nomenclature

- Vertebral Fracture Assessment (VFA) is the correct term to denote densitometric spine imaging performed for the purpose of detecting vertebral fractures.

Indications for VFA

- Lateral Spine imaging with Standard Radiography or Densitometric VFA is indicated when T-score is < -1.0 and of one or more of the following is present:
  - Women age ≥ 70 years or men ≥ age 80 years
  - Historical height loss > 4 cm (>1.5 inches)
  - Self-reported but undocumented prior vertebral fracture
  - Glucocorticoid therapy equivalent to ≥ 5 mg of prednisone or equivalent per day for ≥ 3 months

Methods for Defining and Reporting Fractures on VFA

- The methodology utilized for vertebral fracture identification should be similar to standard radiological approaches and be provided in the report.
- Fracture diagnosis should be based on visual evaluation and include assessment of grade/severity. Morphometry alone is not recommended because it is unreliable for diagnosis.
- The Genant visual semi-quantitative method is the current clinical technique of choice for diagnosing vertebral fracture with VFA.
- Severity of deformity may be confirmed by morphometric measurement if desired.

Indications for Following VFA With Another Imaging Modality

- The decision to perform additional imaging must be based on each patient's overall clinical picture, including the VFA result.
- Indications for follow-up imaging studies include:
  - Two or more mild (grade 1) deformities without any moderate or severe (grade 2 or 3) deformities
  - Lesions in vertebrae that cannot be attributed to benign causes
  - Vertebral deformities in a patient with a known history of a relevant malignancy
  - Equivocal fractures
  - Unidentifiable vertebrae between T7-L4
  - Sclerotic or lytic changes, or findings suggestive of conditions other than osteoporosis

Note: VFA is designed to detect vertebral fractures and not other abnormalities.

Baseline DXA Report: Minimum Requirements

- Demographics (name, medical record identifying number, date of birth, sex).
- Requesting provider.
- Indications for the test.

- Manufacturer and model of instrument used
- Technical quality and limitations of the study, stating why a specific site or ROI is invalid or not included.
- BMD in g/cm2 for each site.
- The skeletal sites, ROI, and, if appropriate, the side, that were scanned.
- The T-score and/or Z-score where appropriate.
- WHO criteria for diagnosis in postmenopausal females and in men age 50 and over.
- Risk factors including information regarding previous non-traumatic fractures.
- A statement about fracture risk. Any use of relative fracture risk must specify the population of comparison (e.g., young- adult or age-matched). The ISCD favors the use of absolute fracture risk prediction when such methodologies are established.
- A general statement that a medical evaluation for secondary causes of low BMD may be appropriate.
- Recommendations for the necessity and timing of the next BMD study.

Follow-Up DXA Report

- Statement regarding which previous or baseline study and ROI is being used for comparison.
- Statement about the LSC at your facility and the statistical significance of the comparison.
- Report significant change, if any, between the current and previous study or studies in g/cm2 and percentage.
- Comments on any outside study including manufacturer and model on which previous studies were performed and the appropriateness of the comparison.
- Recommendations for the necessity and timing of the next BMD study.

DXA Report: Optional Items

- Recommendation for further non-BMD testing, such as X-ray, magnetic resonance imaging, computed tomography, etc.
- Recommendations for pharmacological and non-pharmacological interventions.
- Addition of the percentage compared to a reference population.
- Specific recommendations for evaluation of secondary osteoporosis.

DXA Report: Items That Should not be Included

- A statement that there is bone loss without knowledge of previous bone density.
- Mention of “mild,” “moderate,” or “marked” osteopenia or osteoporosis.

- Separate diagnoses for different ROI (e.g., osteopenia at the hip and osteoporosis at the spine).
- Expressions such as “She has the bones of an 80-year-old,” if the patient is not 80 years old.
- Results from skeletal sites that are not technically valid.
- The change in BMD if it is not a significant change based on the precision error and LSC.

Components of a VFA Report

- Patient identification, referring physician, indication(s) for study, technical quality, and interpretation.
- A follow-up VFA report should also include comparability of studies and clinical significance of changes, if any.
- VFA reports should comment on the following
    - Unevaluable vertebrae
    - Deformed vertebrae, and whether or not the deformities are consistent with vertebral fracture
    - Unexplained vertebral and extra-vertebral pathology
- Optional components include fracture risk and recommendations for additional studies.

Trabecular Bone Score (TBS)

- **TBS is associated with vertebral, hip and major osteoporotic fracture risk in postmenopausal women.**
- **TBS is associated with hip fracture risk in men over the age of 50 years.**
- **TBS is associated with major osteoporotic fracture risk in men over the age of 50 years.**
- **TBS should not be used alone to determine treatment recommendations in clinical practice.**
- **TBS can be used in association with FRAX and BMD to adjust FRAX-probability of fracture in postmenopausal women and older men.**
- **TBS is not useful for monitoring bisphosphonate treatment in postmenopausal women with osteoporosis.**
- **TBS is associated with major osteoporotic fracture risk in postmenopausal women with type II diabetes.**

Hip Geometry

- **Hip axis length (HAL) derived from DXA is associated with hip fracture risk in postmenopausal women.**
- **The following hip geometry parameters derived from DXA (CSA, OD, SM, BR, CSMI, NSA) should not be used to assess hip fracture risk.**

- **Hip geometry parameters derived from DXA (CSA, OD, SM, BR, CSMI, HAL, NSA) should not be used to initiate treatment.**
- **Hip geometry parameters derived from DXA (CSA, OD, SM, BR, CSMI, HAL, NSA) should not be used for monitoring.**

General Recommendations for Non Central DXA Devices: QCT, pQCT, QUS, and pDXA

The following general recommendations for QCT, pQCT, QUS, and pDXA are analogous to those defined for central DXA technologies. Examples of technical differences amongst devices, fracture prediction ability for current manufacturers and equivalence study requirements are provided in the full text documents printed in the *Journal of Clinical Densitometry*.

- Bone density measurements from different devices cannot be directly compared.
- Different devices should be independently validated for fracture risk prediction by prospective trials, or by demonstration of equivalence to a clinically validated device.
- T-scores from measurements other than DXA at the femur neck, total femur, lumbar spine, or one-third (33%) radius cannot be used according to the WHO diagnostic classification because those T-scores are not equivalent to T-scores derived by DXA.
- Device-specific education and training should be provided to the operators and interpreters prior to clinical use.
- Quality control procedures should be performed regularly.

Baseline Non Central DXA Devices (QCT, pQCT, QUS, pDXA) Report: Minimum Requirements

- Date of test
- Demographics (name, date of birth or age, sex)
- Requesting provider
- Names of those receiving copy of report
- Indications for test
- Manufacturer, and model of instrument and software version
- Measurement value(s)
- Reference database
- Skeletal site/ROI
- Quality of test
- Limitations of the test including a statement that the WHO diagnostic classification cannot be applied to T-scores obtained from QCT, pQCT, QUS, and pDXA (other than one-third (33%) radius) measurements
- Clinical risk factors
- Fracture risk estimation

- A general statement that a medical evaluation for secondary causes of low BMD may be appropriate
- Recommendations for follow-up imaging

Note: A list of appropriate technical items is provided in the QCT and pQCT sections of the full text documents printed in the *Journal of Clinical Densitometry.*

Non Central DXA Devices (QCT, pQCT, QUS, pDXA) Report: Optional Items

- Report may include the following optional item:
    - Recommendations for pharmacological and non-pharmacological interventions.

QCT and pQCT

- **Acquisition**
    - With single-slice QCT, L1-L3 should be scanned; with 3D QCT, L1-L2 should be scanned.
    - **QCT acquisition of the proximal femur should extend from the femoral head to the proximal shaft.**
    - **For density-based QCT measurements the in-scan calibration phantom can be replaced by asynchronous calibration if scanner stability is maintained.**
    - **Opportunistic CT to screen for patients with low BMD or low bone strength of the spine or proximal femur is possible only if validated machine-specific cutoff values and scanner stability have been established.**
- **Diagnosis**
    - **Femoral neck and total hip T-scores calculated from 2D projections of QCT data are equivalent to the corresponding DXA T-scores for diagnosis of osteoporosis in accordance with the WHO criteria.**
- **Fracture Prediction**
    - Spinal trabecular BMD as measured by QCT has at least the same ability to predict vertebral fractures as AP spinal BMD measured by central DXA in postmenopausal women. There is lack of sufficient evidence to support this position for men.
    - There is lack of sufficient evidence to recommend spine QCT for hip fracture prediction in either women or men.
    - **Total femur trabecular BMD measured by QCT predicts hip fractures as well as hip BMD measured by DXA in postmenopausal women and older men.**
    - pQCT of the forearm at the ultra-distal radius predicts hip, but not spine, fragility fractures in postmenopausal women. There is lack of sufficient evidence to support this position for men.
- **Therapeutic Decisions**

- Central DXA measurements at the spine and femur are the preferred method for making therapeutic decisions and should be used if possible. **Where QCT and DXA are both available and provide comparable information, DXA is preferred to limit radiation exposure.**
    - However, if central DXA cannot be done, pharmacologic treatment can be initiated if the fracture probability, as assessed by QCT of the spine or pQCT of the radius using device specific thresholds, and in conjunction with clinical risk factors, is sufficiently high.
- **Monitoring**
    - Trabecular BMD of the lumbar spine measured by QCT can be used to monitor age-, disease-, and treatment-related BMD changes.
    - **Integral and trabecular BMD of the proximal femur measured by QCT can be used to monitor age- and treatment-related BMD changes.**
    - Trabecular and total BMD of the ultra-distal radius measured by pQCT can be used to monitor age-related BMD changes.
- **Finite Element Analysis (FEA)**
    - **Vertebral strength as estimated by QCT-based FEA predicts vertebral fracture in postmenopausal women.**
    - **Vertebral strength as estimated by QCT-based FEA is comparable to spine DXA for prediction of vertebral fractures in older men.**
    - **Femoral strength as estimated by QCT-based FEA is comparable to hip DXA for prediction of hip fractures in postmenopausal women and older men.**
    - **FEA cannot be used to diagnose osteoporosis using the current WHO T-score definition.**
    - **Vertebral or femoral strength as estimated by QCT-based FEA can be used to initiate pharmacologic treatment using validated thresholds and in conjunction with clinical risk factors.**
    - **Vertebral or femoral strength as estimated by QCT-based FEA can be used to monitor age- and treatment-related changes.**
- Reporting
    - For QCT using whole body CT scanners the following additional technical items should be reported:
        - Tomographic acquisition and reconstruction parameters
        - kV, mAs
        - Collimation during acquisition
        - Table increment per rotation
        - Table height
        - Reconstructed slice thickness, reconstruction increment
        - Reconstruction kernel

- For pQCT using dedicated pQCT scanners, the following additional technical items should be reported:
  - Tomographic acquisition and reconstruction parameters
  - Reconstructed slice thickness
  - Single / multi-slice acquisition mode
  - Length of scan range in multi-slice acquisition mode

QUS

- Acquisition
  - The only validated skeletal site for the clinical use of QUS in osteoporosis management is the heel.
- Fracture Prediction
  - Validated heel QUS devices predict fragility fracture in postmenopausal women (hip, vertebral, and global fracture risk) and men over the age of 65 (hip and all non-vertebral fractures), independently of central DXA BMD.
  - Discordant results between heel QUS and central DXA are not infrequent and are not necessarily an indication of methodological error.
  - Heel QUS in conjunction with clinical risk factors can be used to identify a population at very low fracture probability in which no further diagnostic evaluation may be necessary. (Examples of device-specific thresholds and case findings strategy are provided in the full text documents printed in the *Journal of Clinical Densitometry*.)
- Therapeutic Decisions
  - Central DXA measurements at the spine and femur are preferred for making therapeutic decisions and should be used if possible. However, if central DXA cannot be done, pharmacologic treatment can be initiated if the fracture probability, as assessed by heel QUS, using device specific thresholds and in conjunction with clinical risk factors, is sufficiently high. (Examples of device-specific thresholds are provided in the full text documents printed in the *Journal of Clinical Densitometry*.)
- Monitoring
  - QUS cannot be used to monitor the skeletal effects of treatments for osteoporosis.

pDXA

- Fracture Prediction
  - Measurement by validated pDXA devices can be used to assess vertebral and global fragility fracture risk in postmenopausal women, however its vertebral fracture predictive ability is weaker

than central DXA and heel QUS. There is lack of sufficient evidence to support this position for men.
    - Radius pDXA in conjunction with clinical risk factors can be used to identify a population at very low fracture probability in which no further diagnostic evaluation may be necessary. (Examples of device-specific thresholds and case findings strategy are provided in the full text documents printed in the *Journal of Clinical Densitometry*.)
- Diagnosis
    - The WHO diagnostic classification can only be applied to DXA at the femur neck, total femur, lumbar spine and the one-third (33%) radius ROI measured by DXA or pDXA devices utilizing a validated young-adult reference database.
- Therapeutic Decisions
    - Central DXA measurements at the spine and femur are the preferred method for making therapeutic decisions and should be used if possible. However, if central DXA cannot be done, pharmacologic treatment can be initiated if the fracture probability, as assessed by radius pDXA (or DXA) using device specific thresholds and in conjunction with clinical risk factors, is sufficiently high. (Examples of device-specific thresholds are provided in the full text documents printed in the *Journal of Clinical Densitometry*.)
- Monitoring
    - pDXA devices are not clinically useful in monitoring the skeletal effects of presently available medical treatments for osteoporosis.

Body Composition

- Indications
    - DXA total body composition with regional analysis can be used in the following conditions:
        - In patients living with HIV to assess fat distribution in those using anti-retroviral agents associated with a risk of lipoatrophy (currently stavudine [d4T] and zidovudine [ZDV, AZT]).
        - In obese patients undergoing bariatric surgery (or medical, diet, or weight loss regimens with anticipated large weight loss) to assess fat and lean mass changes when weight loss exceeds approximately 10%. The impact on clinical outcomes is uncertain.
        - In patients with muscle weakness or poor physical functioning to assess fat and lean mass. The impact on clinical outcomes is uncertain.
    - Pregnancy is a contraindication to DXA body composition. Limitations in the use of clinical DXA for total body composition or bone mineral density are weight over the table limit, recent

administration of contrast material and/or artifact. Radiopharmaceutical agents may interfere with accuracy of results using systems from some DXA manufacturers.

- Acquisition
  - No phantom has been identified to remove systematic differences in body composition when comparing in-vivo results across manufacturers.
  - An in-vivo cross-calibration study is necessary when comparing in-vivo results across manufacturers.
  - Cross-calibrating systems of the same make and model can be performed with an appropriate whole body phantom.
  - Changes in body composition measures can be evaluated between two different systems of the same make and model if the systems have been cross-calibrated with an appropriate total body phantom.
  - When changing hardware, but not the entire system, or when replacing a system with the same technology (make and model), cross-calibration should be performed by having one technologist do 10 whole body phantom scans, with repositioning, before and after hardware change. If a greater than 2% difference in mean percent fat mass, fat mass or lean mass is observed, contact the manufacturer for service/correction.
  - No total body phantoms are available at this time that can be used as absolute reference standards for soft-tissue composition or bone mineral mass.
  - The Quality Control (QC) program at a DXA body composition facility should include adherence to manufacturer guidelines for system maintenance. In addition, if not recommended in the manufacturer protocol, the following QC procedures are advised:
    - Perform periodic (at least once per week) body composition phantom scans for any DXA system as an independent assessment of system calibration.
    - Plot and review data from calibration and body composition phantom scans.
    - Verify the body composition phantom mean percent fat mass and tissue mass after any service performed on the densitometer.
    - Establish and enforce corrective action thresholds that trigger a call for service.
    - Maintain service logs.
    - Comply with radiation surveys and regulatory government inspections, radiation surveys and regulatory requirements.
  - Consistent positioning and preparation (e.g. fasting state, clothing, time of day, physical activity, empty bladder) of the patient is important for precise measures.
  - Positioning of the arms, hands, legs and feet whenever possible should be according to the NHANES method (palms down isolated

    from the body, feet neutral, ankles strapped, arms straight or slightly angled, face up with neutral chin).
    - "Offset-scanning" should be used in patients who are too wide to fit within the scan boundaries, using a validated procedure for a specific scanner model.
    - Every technologist should perform an in-vivo precision assessment for all body composition measures of interest using patients who are representative of the clinic's patient population.
    - The minimum acceptable precision for an individual technologist is 3%, 2% and 2% for total fat mass, total lean mass, and percent fat mass, respectively.
    - Consistently use manufacturer's recommendations for ROI placement.
    - Consistently use manufacturer's recommendations for artifact removal.
- Analysis and Reporting
    - For adults total body (with head) values of BMI, BMD, BMC, total mass, total lean mass, total fat mass, and percent fat mass should appear on all reports.
    - Total Body BMC as represented in the NHANES 1999-2004 reference data should be used when using DXA in 4-compartment models.
    - DXA measures of adiposity and lean mass include visceral adipose tissue (VAT), appendicular lean mass index (ALMI: appendicular lean mass/ht^2), android/gynoid percent fat mass ratio, trunk to leg fat mass ratio, lean mass index (LMI: total lean mass/ht^2), fat mass index (FMI: fat mass/ht^2) are optional. The clinical utility of these measures is currently uncertain.
    - When comparing to the US population, the NHANES 1999-2004 body composition data are most appropriate for different races, both sexes, and for ages from 8 to 85 years. [Note: Reference to a population does not imply health status.]
    - Both Z-scores and percentiles are appropriate to report if derived using methods to adjust for non-normality.
    - The use of DXA adiposity measures (percent fat mass or fat mass index) may be useful in risk-stratifying patients for cardio-metabolic outcomes. Specific thresholds to define obesity have not been established.
    - "Low lean mass" could be defined using appendicular lean mass divided by height squared (ALM/$height^2$) with Z-scores derived from a young adult, race, and sex-matched population. Thresholds for low lean mass from consensus guidelines for sarcopenia await confirmation.

Glossary

ALMI – appendicular lean mass index

BMC – bone mineral content

BMD – bone mineral density **(equivalent to areal BMD, aBMD)**

BMI – body mass index

BR – buckling ratio

CSA – Cross Sectional Area

CSMI – cross-sectional moment of inertia

DXA – dual-energy X-ray absorptiometry

FEA – Finite element analysis

FMI – fat mass index

HAL – hip axis length

ISCD – International Society for Clinical Densitometry

LMI – lean mass index

LSC – least significant change

NHANES III – National Health and Nutrition Examination Survey III

NSA – neck shaft angle

OD – outer diameter

PA – posterior anterior

pDXA – peripheral dual-energy x-ray absorptiometry

pQCT – peripheral quantitative computed tomography

QC – quality control

QCT – quantitative Computed Tomography

QUS – quantitative Ultrasound

ROI – region(s) of interest

SM – section modulus

SSI - strain strength index

TBLH – total body less head

TBS – trabecular bone score

VAT – visceral adipose tissue

VFA – Vertebral Fracture Assessment

vBMD – volumetric BMD

WHO – World Health Organization

© Copyright ISCD, June 2015. Supersedes all prior “Official Positions” publications.

UPDATED: June 16, 2015

Last modified: June 16, 2015
