# Supplementary material for: Case report: lady with bone pains for 5 years—parathyroid carcinoma
Source: BMC Res Notes. 2018 Aug 29;11:617. doi: 10.1186/s13104-018-3711-0 (PMC6114890; doi:10.1186/s13104-018-3711-0)
Supplement: Supplementary file 2 — Additional file 2. Para cancer patient perspective. Title of Data: Patient Perspective. Description of Data: describes the patient’s view on how challenging the diagnosis of parathyroid cancer had been for her and her family, and the toll it took on them. She emphasizes the importance of creating awareness about the condition amongst general practitioners to expedite early referral to concerned speciality. [file 13104_2018_3711_MOESM2_ESM.docx]

**Patient Perspective**

I had suffered for years. There was a point when I thought that I should just stop seeking help for my condition. I had done everything the doctors advised, including the inconvenient procedures, like bone-marrow examinations. I went for that *two* times. Still no definite answers were provided. If it had not been for the continuous support and encouragement from my family, I would not have returned for any follow up visits, not even years later. I am very grateful to Allah (God) that we eventually went to the right place, where my condition was finally diagnosed. I would urge my care givers to instill and spread awareness of the condition to general practitioners, as it is they that are in the front line dealing with all types of patients. I am fully aware that what I had, that is, **cancer** of **parathyroid** is extremely rare, perhaps 1%, perhaps less of entire population. However, when this kind of condition hits oneself or a loved one, it is **100%** for that person.
